# The Microbiome of Animals: Implications for Conservation Biology

**DOI:** 10.1155/2016/5304028

**Published:** 2016-04-18

**Authors:** Simon Bahrndorff, Tibebu Alemu, Temesgen Alemneh, Jeppe Lund Nielsen

**Affiliations:** ^1^Section of Biology and Environmental Science, Department of Chemistry and Bioscience, Aalborg University, Fredrik Bajers Vej 7H, 9220 Aalborg East, Denmark; ^2^Department of Environmental Health Science and Technology, College of Health Science, Jimma University, Jimma, Ethiopia

## Abstract

In recent years the human microbiome has become a growing area of research and it is becoming clear that the microbiome of humans plays an important role for human health. Extensive research is now going into cataloging and annotating the functional role of the human microbiome. The ability to explore and describe the microbiome of any species has become possible due to new methods for sequencing. These techniques allow comprehensive surveys of the composition of the microbiome of nonmodel organisms of which relatively little is known. Some attention has been paid to the microbiome of insect species including important vectors of pathogens of human and veterinary importance, agricultural pests, and model species. Together these studies suggest that the microbiome of insects is highly dependent on the environment, species, and populations and affects the fitness of species. These fitness effects can have important implications for the conservation and management of species and populations. Further, these results are important for our understanding of invasion of nonnative species, responses to pathogens, and responses to chemicals and global climate change in the present and future.

## 1. Introduction

The microbiomes, including bacteria, fungi, and viruses, live within and upon all organisms and have become a growing area of research. With the advances of new technologies it is now possible to entangle complex microbial communities found across animal kingdoms.

Recent advances in molecular biology have provided new possibilities to investigate complex microbial communities and it has become clear that the vast majority of bacteria living in/on other animals cannot be cultured. It is now commonly accepted that at least 80% of the total bacterial species in the human gut cannot yet be cultured [1, 2].

High-throughput DNA sequencing approaches provide an attractive and cost-effective approach to investigate the composition and functions of the host microbiome. The culture-independent analysis of the host microbiome can be obtained by either metagenomic approaches or amplicon sequencing using specific marker genes. Amplicon sequencing provides a targeted version of metagenomics with a specific genetic region shared by the community members of interest. The amplified fragments derive from universal primers and are usually assumed to produce sequence read abundance that reflects the genetic diversity in the studied sample and hence sequence read abundance should reflect the genetic diversity in the studied sample. The amplified fragment typically contains phylogenetic or functional information, such as the 16S ribosomal RNA gene. 16S rRNA gene sequences are well studied and provide excellent tools for microbial community analysis [3], but other functional marker genes can also be used [4]. Subsequent taxonomy profiling of the entire microbial communities is conducted by comparisons to reference sequences or by* de novo *clustering of specific regions of sequences. Functional profiling of metagenomics is more challenging since major parts of the metagenomic data remain insufficiently characterized and frequently samples are contaminated by host DNA or traces from the diet. Compared to both culture-dependent and more traditional molecular approaches such as sequencing of clone libraries and DGGE, amplicon sequencing approaches allow a more in depth analysis of the complete microbiome and are less restricted to the number of samples to be investigated. For further technical details see, for example, Caporaso et al. [3].

## 2. The Microbiome of Animals

The Human Microbiome Project (HMP) [1] was initiated in 2007 and with this it has become clear that the human microbiome is highly diverse and complex. The number of microorganisms sharing the human body is thought to outnumber human cell numbers by a factor of ten and the combined microbiome usually contains 100x more genes than its host. The microbiome also plays a major role in human health [5] and both composition and alterations in the microbiome have been found associated with diabetes, inflammatory bowel disease, obesity, asthma, rheumatoid arthritis, and susceptibility to infections [6–11].

In recent years the microbiome of a number of vertebrate nonhuman species has been sequenced including livestock [12, 13] and wildlife species such as the Tasmanian devil [14], red panda [15], giant panda [16], black howler monkey [17], and koala [18].

Insects are the most diverse and abundant groups of animals on earth [19] and have colonized many different habitats. It is therefore not surprising that insect species are also inhabited by large and diverse microbial communities playing a pivotal role for insect biology. Many insect species are inhabited by a large and diverse assembly of microorganisms, where especially the microbial communities in the intestinal tract have received much attention [20–22]. Some insect species show a much more diverse microbiome compared to other insect species. For example, the microbiomes of some synanthropic flies, such as the green bottle fly, show high diversity compared to other species such as fruit flies or mosquitoes [23–25]. The high species richness could reflect the lifestyle of synanthropic flies, for example, breeding and living by animal manure, bedding, and/or decaying organic matter rich in microorganisms.

The microbiome of other groups of invertebrates has also been established although for a limited number of species. Studies have compared the microbiome of different species of marine invertebrates with or without photosynthetic symbionts including five families of marine invertebrates [26]. Marine species of commercial interest such as oysters have also been addressed [27].

The microbes of soil invertebrates have received some attention. The gut microbes of soil animals play an indispensable role in the digestion of food and are of ecological importance in the global carbon cycle. Recently, research reported that like that of terrestrial insects some soil invertebrates such as collembolans, earthworms, and nematodes contain a rich microbiome and putative symbionts [28–30]. Further, results have shown how differences in diet among earthworm ecological groups lead to the establishment of different bacterial communities [28]. Moreover, perturbation of the soil ecosystem could impact earthworm gut wall-associated bacterial community composition and hence earthworm ecology and functioning. Even though the microbial community in invertebrates like that of collembolans and earthworms is not fully addressed, there is convincing evidence that intestinal communities can contribute to the degradation of recalcitrant biological materials such as chitin and lignocellulose [28, 29, 31].

## 3. Factors Affecting the Animal Microbiome and the Biological Significance

To begin with all microorganisms were seen as pathogens causing infectious diseases to the host. The host immune system of eukaryotes was built to eliminate these intruders, but at the same time tolerating its own molecules. However, we now know that the association between eukaryotic hosts and the microorganisms is far more complex. With the advances in molecular biology, such as next generation sequencing, it is now possible more specifically to address the association between a host and its microbiome. In animals the association between the host and its microbiome can take many forms and includes symbiotic and pathogenic associations [20]. Symbiotic microbiomes can be beneficial to the hosts in many ways, including dietary supplementation, host immune system, and social interactions [21, 32]. In many insects, the gut symbionts are essential for survival and development and suggest the presence of a core microbiome [33]. The symbionts need not to be completely dependent on the host and animal-microbial interactions can be flexible and facultative and the host can carry different symbionts at different times [20]. The association between the host and the microbiome is also affected by a large number of abiotic and biotic factors and can involve the immune system, nutrition, reproduction, communication, and many other systems of the host [2, 34–36].

The number of studies addressing the role of the microbiome on animal health is limited and almost entirely restricted to human studies. However, a large number of studies have addressed the role of single bacterial symbionts on animal fitness, where especially insect species have received much attention [37–39]. There is now a growing interest in understanding what factors can affect the microbiome of animals in order to understand how fitness is affected and to explain differences between ecosystems, species, and/or populations. The composition of the bacterial communities of animals including invertebrates and vertebrates seems to be shaped by multiple factors, such as the host genotype [22, 23, 40, 41], diet [17, 34, 37, 42], life stage [43], laboratory rearing [34, 43, 44], and the ecological and physiological conditions of, for example, the gut of the insect [22]. Further, recent studies have proposed that the microbiome impacts the nutritional supplementation, tolerance to environmental perturbations, and maintenance and/or development of the immune system [20].

Some invertebrates lack the complexity and diversity of associations with microorganisms. Such insect model systems allow investigations that aim to understand the contribution of specific bacteria and the entire microbiome towards host physiological processes. For example,* Drosophila melanogaster* provide a promising model system to address some of these issues and for this species it is possible to rear axenic flies. Next generation sequencing approaches can provide an in-depth analysis of the functional roles of specific groups of bacteria and the entire microbiome on the fitness of the host. Results on* D. melanogaster* have shown how the microbiota affects developmental rate and changes metabolic rates and carbohydrate allocation under laboratory conditions [32]. Similarly functional analysis of the microbiome of ants also suggests large capacity to degrade cellulose [45] and that metabolic functions of microbes in herbivorous species play a role in fixing, recycling, or upgrading nitrogen [46]. Hypothesis has also been proposed to describe that gut microbiomes might facilitate insect herbivory and that variation in the ability to consume chemically defended plants can be partly explained by variation in the gut microbiome [47].

Recent studies have highlighted the importance of the microbiome not only in shaping the immune system but also in the context of host pathogen transmission processes (for reviews see [20, 48]). An example hereof is that the success of malaria infections is not only influenced by the mosquito innate immune responses and genetics but also affected by the composition of the gut microbiota and is in fact one of the major components affecting the outcome of mosquito infections [24]. Studies have also suggested that abiotic factors can affect the microbiome of disease vectors and thus vector competence of the host [25, 35]. Similarly the epidemics of human pathogens transmitted by insect vectors correlate with environmental factors [49, 50] suggesting that the vector competence of insect vectors is affected both indirectly and directly by environmental factors [35, 51, 52].

The recent interest in the importance of the microbiome on tolerance to environmental perturbations [38, 39] has revealed the presence of single bacterial species and mainly endosymbionts with large impact on, for example, temperature tolerance (for review see [39]). Temperature can affect the host directly or indirectly through either abundance of the symbiont or efficiency of transmission to the offspring [53–55]. At present it is unclear to what degree single strains of bacteria play a dominant role in tolerance to environmental factors or if interactions between bacteria of the microbiome are dominant. The recent advances in molecular biology and implementation of statistical analysis allow more specific hypothesis to be tested on effects of the microbiome on tolerance to, for example, environmental stress.

## 4. Conservation and Implications for Conservation

Changes in the microbial community have been shown to affect fitness of humans and other species as described above. However, the implications of changes in the microbiome for animal conservation have only been addressed in a limited number of studies even though the implications are many.

Several studies using next generation sequencing approaches have addressed the comparison of the microbiome of laboratory populations or individuals kept in captivity with that of wild animals [14, 15, 18, 34, 44] or of single species in habitats influenced by different degrees of human behavior [17]. Results show that species across taxa living under laboratory conditions or affected by habitat fragmentation show less diverse microbiomes compared to wild species. Thus species are jeopardized not only directly by degraded habitats with reduced resource availability but also indirectly through diminished microbiomes. It is thus essential that future studies address the microbiome and how habitat fragmentation impacts the microbiome in different species and how species with less diverse microbiomes perform under these conditions.

It is essential that we address the importance of the microbiome of other species rather than humans and the impact it has on their health status. For larger species such as primates this can be difficult and often only correlative evidence exists or can be achieved through a functional annotation of the microbiome [14, 17]. For example, in a study by Amato and coworkers [17] it was shown that beneficial fermenters, acetogens, and methanogen bacteria were more abundant in black howler monkeys inhabiting evergreen rainforest compared to individuals from fragmented habitats. The latter group also contained higher numbers of sulfate-reducing bacteria producing undesirable end products such as H_2_S. This strongly suggests that habitat fragmentation will affect not only the microbiome of the host but also host fitness.

Similarly, keeping animals under captivity and maintaining breeding populations are likely to affect animal microbiomes. This is often undertaken in order to protect or increase abundance of rare species aiming at releasing species into the wild again. However, if the microbiomes of the individuals being released are affected, this is likely also to affect fitness compared to that of wild individuals and will subsequently reduce the probability of successful reintroduction into the wild. This is supported by studies on humans and mice where results have shown that obesity causes shifts in gut microbiome composition [6, 56]. Similar nutritional conditions could be expected for individuals kept in captivity. Molecular approaches allow researchers to establish entire microbiomes of animals and thus also test if, for example, it is possible to acclimate animals before being released into the wild. Optimizing environmental conditions of species in captivity could potentially ensure successful management and reintroduction.

It has been suggested that engineering microbiomes can be used to improve plant and animal health [57]. How this can be incorporated into conservation is unclear. It is standard to employ basic principles of genetics into breeding strategies for endangered species in zoos or captivity, but the microbiomes evolutionary potential has been ignored also in conservation biology.

Inbreeding has been suggested to affect the demography and persistence of natural populations and play an important role in conservation biology [58]. Recent work shows that inbreeding depression in bird and mammal populations significantly affects birth weight, survival, reproduction, and resistance to disease, predation, and environmental stress [59]. Inbreeding depression is expected to change the proportions of homozygotes and thus also heterozygotes. Consequently recessive deleterious mutations are likely to be expressed. As fitness of animal populations is expected to be affected by genotype of the host and the microbiome and interaction between the two it is also likely that the microbiome will be affected by inbreeding depression either directly or through interaction with the genotype of the host, not only because the genepool is diminished but also because of a compromised immune system.

Microbiome analysis of wild populations has shown that the microbiome is dependent on the surrounding habitats as discussed above. This information might be used as a sensitive screening tool to establish populations affected by habitat fragmentation [17] and possibly also the effect of inbreeding. The strong signal from the diet [17, 34, 37, 42] suggests that the microbiome can also be used as a screening tool of diet preferences and to protect critical food resources or habitats for endangered species. However, it is essential that we fully understand the temporal and spatial changes in the microbiome if we are to use it as a screening tool.

The microbiome can provide protection of the host from pathogens either through stimulation of the immune system or through competitive exclusion. However, when animals are compromised or exposed to unfavorable environmental conditions the symbionts themselves can act as opportunistic pathogens [2, 27] or not provide the same degree of protection. There are examples of how environmental conditions can affect the microbiome of invertebrates. For example, studies have shown how changes in temperature have caused shifts from mutualistic to pathogen dominated communities in corals [60]. In oysters temperatures over 20°C can cause summer mortalities, but temperatures as low as 14°C will promote development of brown ring disease in clams [61, 62]. This is important in conservation biology given the fact that species and populations are or will be exposed to changes in climate under the future climate scenarios. Host species will thus be exposed to not only the direct effects of changes in, for example, temperature but also indirect effects due to change in abundance or species composition of the microbiome. These changes can again lead to direct fitness effects on the host or indirect effects through changes or modification of the immune response. The microbiome could potentially also allow organisms to respond on a short timescale and cope with, for example, changes in climate. In particular, for species with a long generation time populations might not be able to adapt to fast changes in climate. However, bacteria with a short generation time can adapt on a shorter timescale compared to the host and may provide fitness advantages that allow the host to cope with changes in climate. Future studies should more specifically test if and how the microbiome affects animals ability to respond to a changing environment. Such plastic responses can have important implications for persistence of species or populations at risk in a fluctuating environment.

Differences in microbiomes may affect invasions. For example, the interactions between native and nonnative of closely related species may be affected by the transmission of bacteria. This also appears to be associated with another emerging type of invasion, the transmission of infectious diseases of wild animals to humans [63]. Such transmission may be associated with factors including changes in human and nonhuman microbiomes. These interactions also have important implications for the conservation and management of different species within the environment. Some studies have addressed the microbiome of invasive species and also compared populations originating from the species native region with that of invasive regions [64, 65]. For the invasive snail,* Achatina fulica *results showed a highly diverse microbiome and functional analysis revealed a variety of microbial genes encoding enzymes, which is in agreement with the wide-ranging diet of this species [65]. Interestingly in another study comparing the microbiome of the soybean aphid,* Aphis glycines* from populations of native and invasive regions showed no differences [64]. Future studies should address the importance of the microbiome of invasive species to investigate if single strains of bacteria, the entire microbiome, or their interactions are major determinants for a species ability to establish in a new environment and if invasive microorganisms carried by introduced species affect native species [66].

## 5. Conclusions

Recent advances in molecular biology have given new possibilities to establish complex microbial communities and it has become clear that the vast majority of bacteria living in/on other animals cannot be cultured. One of the most common methods to describe complex microbiomes is the sequencing of the bacterial marker 16S ribosomal RNA (16S rRNA) genes through amplicon sequencing. Studies have shown that the microbiome plays a major role in human health, and in recent years the microbiomes of an increasing number of nonhuman species have been investigated. However, the number of studies addressing the role of the microbiome on animal health still remains limited. Some studies have discussed the role of the microbiome on nutritional supplementation, tolerance to environmental perturbations, and maintenance and development of the immune system. Thus the implications of changes in the microbiome for animal conservation are many although a limited number of studies have addressed this. We suggest that a number of factors relevant in conservation biology could affect the microbiome of animals including inbreeding, habitat fragmentation, change in climate, and effect of keeping animals in captivity. Changes in these factors are thus also likely to affect the fitness of the host both directly and indirectly. With the development of next generation sequencing and functional analysis of microbiomes it has become possible more specifically to test direct hypothesis on the importance of the microbiome in conservation biology.
